# Single and joint toxicity of ethoprophos and bispyribac-sodium to *Oreochromis niloticus*: biochemical and genotoxic responses

**DOI:** 10.1007/s10695-025-01490-2

**Published:** 2025-04-23

**Authors:** Kawther S. EL-Gendy, Eman M. Mosallam, Aya S. Abd El-Kader, Asmaa I. Abdel Monem, Mohamed A. Radwan

**Affiliations:** 1 Pesticide Chemistry and Technology Department, Faculty of Agriculture, Alexandria University, Alexandria, Egypt; 2 Mammalian and Aquatic Toxicology Department, Central Agricultural Pesticide Lab, Agricultural Research Center, Alexandria, Egypt; 3 Animal and Fish Production Department, Faculty of Agriculture, Alexandria University, Alexandria, Egypt

**Keywords:** Pesticide mixture, Ethoprophos, Nile tilapia, Biomarker, Micronucleus test, Neurotoxicity

## Abstract

Pesticides can bioaccumulate in fish tissues, and there are serious concerns around the world about their effects on consumer health. The objective of this study was to highlight the effects of two commercial pesticides, ethoprophos and bispyribac-sodium, commonly used on high-consumption crops in Egypt, either individually or in mixture, on some biomarkers of Nile tilapia, *Oreochromis niloticus*, an important commercial fish species. Low concentrations of ethoprophos (96 µg/L) and bispyribac-sodium (1.28 µg/L) were selected to evaluate the effect of these substances on neurotoxic marker (acetylcholine esterase, AChE), liver function parameters (alkaline phosphatase, ALP; alanine aminotransferase, ALT; and aspartate aminotransferase, AST), and renal function parameters (creatinine and urea), as well as genotoxic marker (micronuclei, MN; and other nuclear abnormalities) during variable periods (7, 14, 21, and 28 days). The results demonstrate that ethoprophos and bispyribac-sodium pose a risk to native freshwater fish by causing detrimental effects. Both compounds, separately and in combination, induced neurotoxicity, hepatorenal biomarkers inductions, and increases in MN frequency and other erythrocytic nuclear abnormalities in a time-dependent manner. Moreover, the mixture displayed both synergistic and antagonistic interactions for examined parameters. This study highlights the importance of using validated biomarkers to monitor fish health, which may be utilized as early alarms of environmental risks.

**Graphical Abstract:**

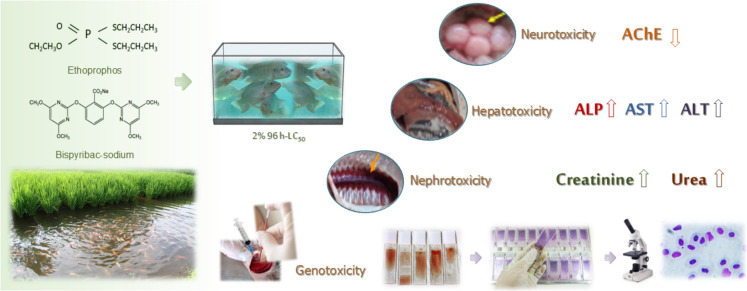

Indiscriminate and injudicious use of pesticides to enhance productivity in agriculture has resulted in contamination of nearby aquatic ecosystems through drainage, spray migration, surface runoff, groundwater seepage, atmospheric deposition, and accidental pesticides spills; therefore, they can be dangerous to a variety of aquatic organisms, mainly fish (Fırat et al. 2011; Weiss et al. 2023). Ethoprophos, an organophosphate compound which acts as acetylcholinesterase inhibitor (Sandoval-Herrera et al. 2019), is utilized pre-planting to control nematodes and soil insects like wireworms. It is used on different crops, ranging from potatoes, bananas, and sugarcane to ornamental plants and tobacco. Bispyribac-sodium, a pyrimidinyl carboxy herbicide, is one of the most used herbicides for controlling sedges, grasses, and broadleaf weeds (European Food Safety Authority 2010) in agricultural land and rice fields around the world, including Egypt. This herbicide prevents cell proliferation by blocking acetolactate synthase, the initial enzyme in the biosynthesis of various amino acids (Shimizu et al. 2002).

Environmental surveys have shown the presence of ethoprophos concentrations in various watercourses, such as 1 µg/L in surface water located in the Caribbean lowlands of Costa Rica (Diepens et al. 2014); 1.076 µg/L along Nile River water, Egypt (Dahshan et al. 2016); and 156.4, 402.7, and 183.5 ng/L in agricultural drainage water from three distinct areas of Lake Burullus, Egypt (El Nahas et al. 2017). Similarly, bispyribac-sodium was detected in different water sources with average concentrations of 3.5 µg/L in irrigated rice growing regions in Brazil (Vieira et al. 2016), 0.02 and 0.06 mg/L in aquatic systems near rice fields in India (Pradhan et al. 2020), and 1.2 to 3.5 µg/mL in agricultural wastewater samples in Egypt (Kamel et al. 2020). Although the environment contains hundreds of persistent pollutants at extremely low concentrations, these pollutants can accumulate in the tissues of aquatic creatures at concentrations that are many times of magnitude larger than those found in the environment (Ray and shaju 2023). For example, El Nahas et al. (2017) determined ethoprophos residue levels in whole tissue of *Oreochromis niloticus* (64.5, 112.1 and 82.5 ng/g) from three distinct areas of Lake Burullus, Egypt. Moreover, ethoprophos concentrations in fish samples obtained from Liaoning Province and the Inner Mongolia autonomous region in Northeast China were 0.126 and 0.085 ng/g, respectively (Fu et al. 2018).

Reportedly, the pesticide amount that actually reaches the target site is less than 0.1% **(**Srivastava et al. 2022**)**; thereby, pesticides continuously reach aquatic ecosystems, are enriched in the aquatic food chain, and may have adverse effects on the nutritional value of fish and on humans through their consumption (Ray and Shaju 2023). Therefore, it is desirable to study the effect of these pesticides on fish, which are a suitable model for monitoring aquatic wastewater quality due to their ability to metabolize and bioaccumulate contaminants **(**Bolognesi and Hayashi 2011**)**. Nile tilapia is one of the aquatic biota species recommended by the US EPA as toxicological test animal (bioindicator) due to its wide distribution in fresh water, high resistance to environmental variables and diseases, rapid growth, high fecundity, easy to maintain in the laboratory and is important in aquaculture (FAO 2020). Additionally, it is of commercial importance in Egypt due to its excellent taste and high nutritional value and provides about 65.15% of the total fish production (GAFRD 2017).

Recently, attempts have been made to create environmental pollution diagnostic methods that utilize biomarkers to detect environmental changes. Due to its greater sensitivity, reduced costs, and increased predictive capacity when compared to chemical monitoring, the biological effect-based assessment of contaminants is particularly significant (Itziou and Dimitriadis 2011; Lionetto et al. 2021). It has long been known that inhibition of AChE activity has been widely applied as a neurotoxic biomarker in aquatic biota (Topal et al. 2017; Ibrahim et al. 2024). Toxicity research investigates a variety of biomarkers to assess a broad spectrum of physiological and metabolic processes influencing target organ recognition and examination of tissue injury. A number of biomarkers, such as hepatotoxicity enzymes like ALP and transaminases (Ogueji et al. 2020; Banaee et al. 2024), and nephrotoxicity markers like creatinine and urea (Ambali et al. 2007; Banaee et al. 2022), can provide more accurate data on recognizing patterns. The micronucleus (MN) test is an important toxicological parameter used to assess the impact of pollutants on aquatic organisms, and this assay is proposed as a technique for detecting mutagenicity (chromosomal damage). As well as it is a more popular, applicable, promising reliable, sensitive, and faster test in genotoxicity studies method in comparison of counting chromosomal aberrations (Dourado et al. 2017). Various studies have shown that the peripheral erythrocytes of fish have a high incidence of micronuclei after exposure to different pesticides under laboratory and field conditions (Hong et al. 2018; El Nahas et al. 2017; Pradhan et al. 2020; Amaeze et al. 2020; Ayanda et al. 2021). There are few studies in the literature that have examined the toxic effects of bispyribac-sodium on *O. niloticus *(Fathy et al. 2019; Saleh et al. 2022). However, no previous research has been conducted on the adverse effects of ethoprophos on *O. niloticus*, and this study fills this knowledge gap.

Most of the times, living organisms are exposed to complex substances including parent compounds and their metabolites, which are very harmful to living beings (EL-Gendy et al. 2023; Banaee et al. 2023, 2024; Kanu et al. 2023). Research on the risk that mixtures of compounds pose to living organisms, particularly aquatic animals, is limited and difficult to predict due to differences in the mode of toxic action, toxicokinetics, and chemical properties of each compound. Studies on the combined effects of many chemicals are necessary due to the proximity to the real environment.

Based on the aforementioned, the present study focuses on the toxicological effects of low concentrations of the two commercial pesticides, ethoprophos (Smart-N, 40% EC) and bispyribac-sodium (Nominee, 2% SL), commonly applied to high-consumed crops in Egypt, either individually or mixture, against Nile tilapia (*O. niloticus*), at different exposure periods (7, 14, 21, and 28 days) on the following biomarkers: acetylcholinesterase, liver function parameters assessed by alkaline phosphatase, aspartate aminotransferase, and alanine aminotransferase activities, and kidney function parameters assessed by urea and creatinine levels, as well as genotoxic marker assessed as micronucleus test.

## Materials and methods

### Chemicals

Ethoprophos (Smart-N 40% EC) was provided by StarChem Company, Egypt. Bispyribac-sodium (Nominee 2% SL) was obtained from Kafr EL Zayat Pesticides and Chemicals Company, Egypt. The remaining chemicals were bought from standard chemical companies and were of the highest possible purity.

### Tested animals

Healthy Nile tilapia fish (*O. niloticus*), weighing 18 ± 6.0 g and 8.6 ± 1.0 cm in length, were attained from a commercial fish farm (Kafr el-Sheikh, Egypt). Fish were incubated in their original field water in glass aquariums of 200 L volume, at a density of 40 individuals per aquarium, in well-aerated condition, and then dechlorinated tap water was gradually added to the acclimatization medium. Fish were adapted in dechlorinated tap water with physicochemical characteristics: pH 7.2 ± 0.2, temperature 25 ± 3 °C, dissolved oxygen content of 8 ± 1 mg/L, total hardness of 61 ± 1.74 mg CaCO_3_/L, and electrical conductivity of 409.07 ± 3.3 µS/cm for 2 weeks. The fish were maintained at a natural photoperiod (12-h light/dark) and fed once daily on commercial fish pellets (19.0% fishmeal, 28.6% soybean meal, 47.4% yellow corn, 3.0% soybean oil, and 2.0% vitamins and minerals). The protocol of the present study was approved by the Institutional Animal Care and Use Committee of the Faculty of Agriculture, Alexandria University, with Code No. AU082209201105.

### Experimental design

The effects of sublethal concentrations of ethoprophos (96 µg/L), bispyribac-sodium (1.28 µg/L), and their mixture on acclimated Nile tilapia were evaluated over variable periods. The pesticide concentration was calculated using the percentage of active ingredients in the commercial pesticide, and the used concentration values, equivalent to 2% of the 96-h LC_50_ were obtained from our previous study (EL-Gendy et al. 2024). A total number of 120 fish were divided into 4 equal groups, the fish of the first group kept as control, while fish of the second, third, and fourth groups were exposed to ethoprophos (96 µg/L), bispyribac-sodium (1.28 µg/L), and mixture of both (ethoprophos 96 µg/L + bispyribac-sodium 1.28 µg/L), respectively. The treated animals were maintained under static renewal conditions, where pesticides and water were completely replaced every 96 h, transferring fish into a freshly prepared toxicants solution. No fish died during exposure to the tested pesticides. Ten fish were picked from each treatment after 7, 14, 21, and 28 days, and used for biochemical and genotoxic studies.

### Sampling

At weekly sampling intervals, the peripheral blood samples were obtained through the gills of each animal with a heparinized capillary tube and used for micronucleus assay. Alongside this, the fish were decapitated; brain, liver, and kidney organs were rapidly removed from each fish and then rinsed thoroughly in cold saline (0.9% NaCL). Each organ sample was weighed and homogenized using a Polytron homogenizer (Tissumizer, Tekmar, Essen, Germany) in ten-volume saline. The homogenate was centrifuged in a Janetzki K23 cooling centrifuge at 5000 × g for 30 min at 4 °C. The resulting supernatant was used to analyze biochemical parameters.

### Biochemical study

#### Determination of neurotoxicity using acetylcholinesterase (AChE) assay

AChE activity was measured according to the procedure of Ellman et al. (1961). The reaction mixture consisted of 0.1 M phosphate buffer, pH 8, 20 µL of brain supernatants, and 100 µL of 0.01 M dithiobisnitrobenzoic acid (DTNB). To this mixture, 20 µL of 0.075 M freshly prepared acetylthiocholine iodide (ATChI) solution was added. The absorbance was measured after 10 min at 412 nm by using a T- 80 + UV/VIS spectrophotometer (spectrometer PG Instruments Ltd., Leicestershire, UK). AChE activity was expressed as µmole/mg protein/min.

#### Determination of liver function parameters

Liver samples were used for determination of ALP, ALT and AST activities. ALP activity was measured by adding 50 µL of liver supernatant to 250 µl of 5.5 mM disodium *p*-nitro phenyl phosphate. Then, after 30 min, the reaction was stopped by adding 0.02 N NaOH and the absorbance was recorded at 405 nm. The activity of ALP is expressed as µmole p-nitrophenol/mg protein/min (Bessey et al. 1946). ALT and AST were investigated spectrophotometrically using commercially available kits (MDSS GmbH, Germany). According to the manufacturer’s instructions, AST activity was evaluated by monitoring the oxaloacetate hydrazone concentration formed with 2, 4-dinitrophenylhydrazine, whereas ALT activity was assayed by monitoring the pyruvate hydrazone concentration formed with 2, 4-dinitrophenylhydrazine. Briefly, the reaction composed of 100 µL of liver supernatant and 1 mL of assay reagents was incubated at RT for 20 min, and then the reaction was stopped by adding 5 ml of 0.4 M NaOH. Absorbance of sample was measured at 546 nm. Activity of transaminases was expressed in terms of U/µg protein/min.

#### Determination of kidney function parameters

Creatinine and urea levels were measured in each kidney supernatants in accordance with the manufacturer’s instructions of commercially available Spectrum Diagnostics kit (MDSS GmbH, Germany). In these assays, 100 µL of creatinine sample or standard was reacted with 1 mL of 25 mM picric acid under alkaline condition to form a yellow–red complex, that read at 492 nm. Whereas, the urea concentration is indirectly based on the measure of colored complex at 578 nm that obtained in an alkaline pH as a result of the hydrolysis of urea sample or standard (10 µL) to produce ammonia.

#### Determination of total protein

Protein content in organ supernatants (brain, liver, or kidney) was evaluated according to the method of Lowry et al. (1951) using bovine serum albumin as standard. Briefly, 5 µL of sample or standard mixed with an alkaline solution (3 mL) was incubated for 10 min, followed by mixing 0.3 mL of Folin-Ciocalteu’s phenol reagent. After 30 min, the intensity of the developed blue color was measured at 750 nm. The results were expressed as milligrams of protein per 5 µL.

#### Determination of chromosome damage using the micronucleus (MN) assay

The MN and other nuclear abnormalities test were performed in blood samples of each animal. Briefly, blood sample obtained through the gills was used immediately to prepare a thin uniform blood smear on a clean glass slide by spreading one drop of blood with another glass slide at 45°. The slides were air-dried for 24 h at RT, fixed in methanol for 10 min, and then left to dry at RT. A 10% aqueous Giemsa solution was applied to the slides and left to dry, then gently rinsed with distilled water and dried. Finally, the slides were examined under a light microscope (OPTIKA B- 150, Italy) using an oil immersion lens at 100 × magnification to observe the nuclear abnormalities. The formation of micronuclei and other nuclear abnormalities was identified using criteria described by Fenech et al. (2003) and Baršienė et al. (2014). Briefly, micronuclei were defined as small, non-refractive, circular, or ovoid chromatin bodies with the same staining pattern and a diameter less than or equal to one-third of the main nucleus. Cells with two nuclei were considered to be binuclear. A small tubular outgrowth of the nuclear membrane and contained euchromatin is known as blebbed. Additionally, the number of micronucleated cells for total 1000 cells were counted per slide and converted to frequencies.

### Interaction of pesticide mixture

The mixture interaction was determined by using an independent effect model, which assumes that the pesticides tested in a mixture have different mechanisms of action on a common biological endpoint. The synergistic or antagonistic interaction between ethoprophos and bispyribac-sodium was determined for biomarkers that showed significant differences between treatments. This was calculated from the ratio of predicted to observed effects caused by exposure to the combination of single compounds, according to Gottardi et al. (2017):

$$Predicted\;effect\;=\:A/C\;\times\:B/C$$

$$Observed\;effect\;=\:M/C$$

$$Ratio\;value\;=\:Predicted\;effect/Observed\;effect$$

where A, B, M, and C are the endpoint values of the organisms exposed to ethoprophos, bispyribac-sodium, mixture, and not exposed, respectively. Synergistic interaction was recorded when the predicted effect was less than the observed effect, whereas antagonism occurred when the predicted effect was greater than the observed effect.

### Statistical analysis

The Student–Newman–Keuls test was used in conjunction with one-way analysis of variance (ANOVA) to create all statistical comparisons. The computer CoStat program, version 6.400, Copyright (CoHort Software: Tucson 1998–2008) was used for analyzing data. Fish were analyzed for level of biochemical markers and frequency of MN between treatments. Level of significance for all tests was set at *p* ≤ 0.05 and data were presented as mean ± respective standard error (SE).

## Results

### AChE activity

**Fig. 1:**
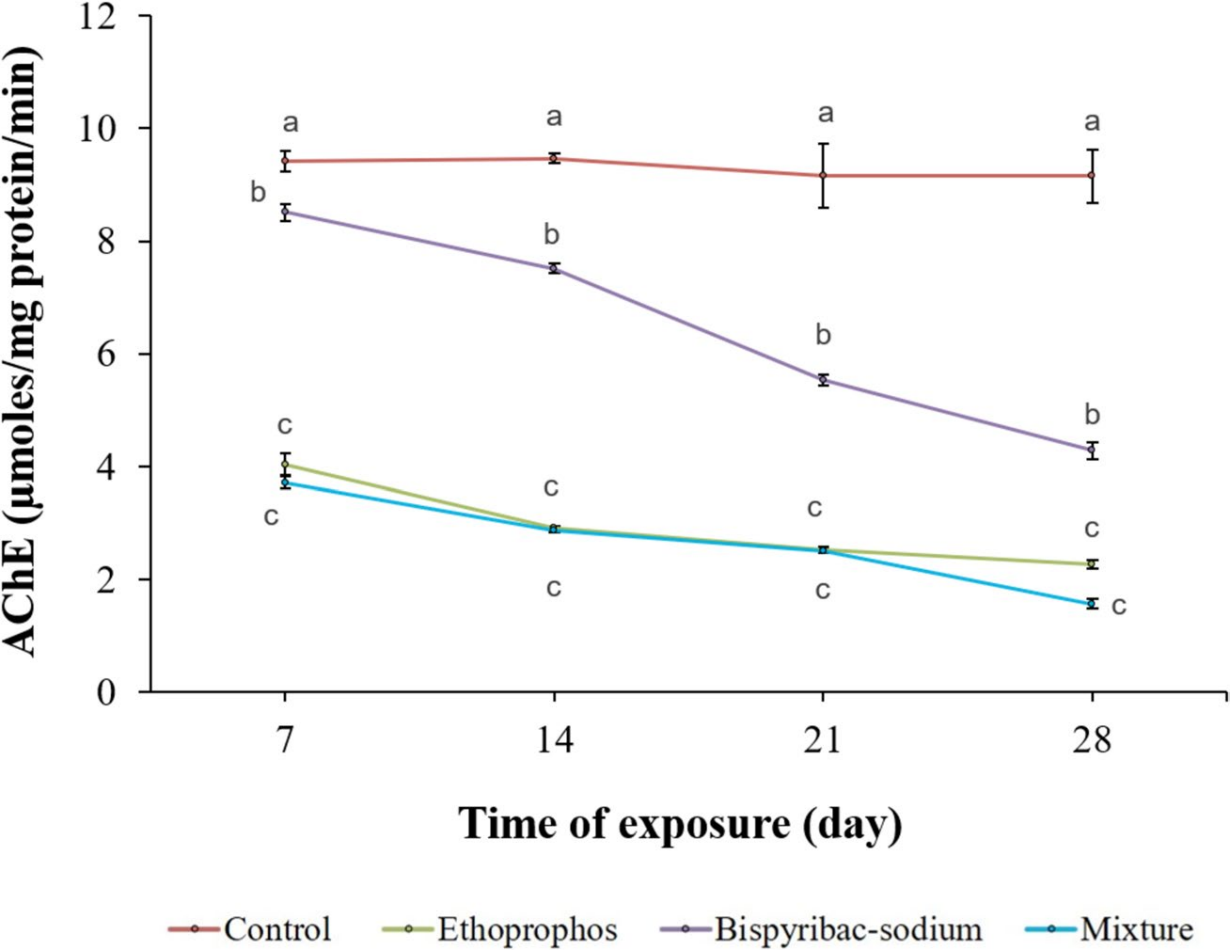
AChE activity in brain tissues of Nile tilapia (*Oreochromis niloticus*) treated with ethoprophos, bispyribac-sodium, and their combination at different exposure times. Values are the mean ± SE; 10 animals (3 replicates per each) were used for each treatment. Superscript different letters (a, b, c) indicate statistically significant differences (*p* ≤ 0.05) between treatments at each exposure time

The current results exhibited a significant (*p* ≤ 0.05) inhibition of AChE activity in the brain of all treated fish at different exposure times when compared to control fish (Fig. 1). The data showed that the binary combination of pesticides had the greatest impact on AChE activity in the fish brain compared to single pesticides, where AChE activity was inhibited by 82.86%, 75.22%, and 53.28% for mixture, ethoprophos, and bispyribac-sodium in the sequence related to the control after 4 weeks of exposure. In addition, the treated fish groups showed statistically considerable time-dependent decrease (*p* ≤ 0.05).

### Liver function markers

**Fig. 2:**
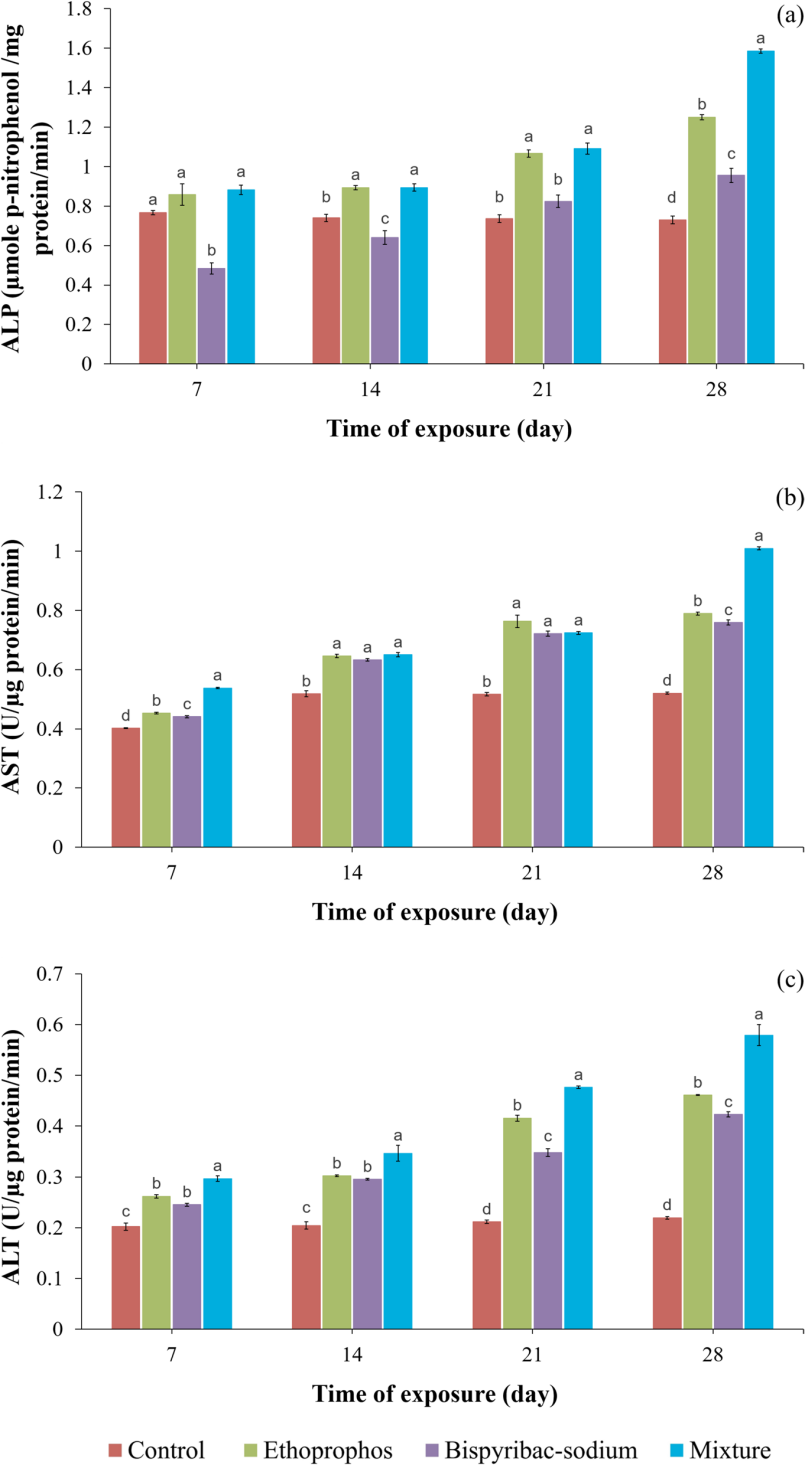
Activity of ALP (**a**), AST (**b**), and ALT (**c**) of Nile tilapia (*Oreochromis niloticus*) treated with ethoprophos, bispyribac-sodium, and their combination at different exposure times. Values are the mean ± SE; 10 animals (3 replicates per each) were used for each treatment. Superscript different letters (a, b, c) indicate statistically significant differences (*p* ≤ 0.05) between treatments at each exposure time

Ethoprophos alone or when combined with bispyribac-sodium considerably (*p* ≤ 0.05) raised the ALP activity after 14, 21, and 28 days of exposure, whereas bispyribac-sodium was significantly increased the ALP level only after 28 days of exposure compared to the normal group (Fig. 2a). The percentage of elevation in ALP level reached 119.18, 63.01, and 21.92 after treatment for 28 days with mixture, ethoprophos, and bispyribac-sodium, respectively.

Transaminase activities in all treatments were also substantially (*p* ≤ 0.05) higher than in the untreated group over 28 days of exposure (Fig. 2b and c). The treatment of Nile tilapia with ethoprophos, bispyribac-sodium, and their mixture for 28 days increased AST activity by 1.52, 1.46, and 1.94 times respectively compared to the control; moreover, the mean values of ALT activity were 0.461, 0.423, and 0.579 U/µg protein/min, respectively.

### Kidney function markers

**Fig. 3:**
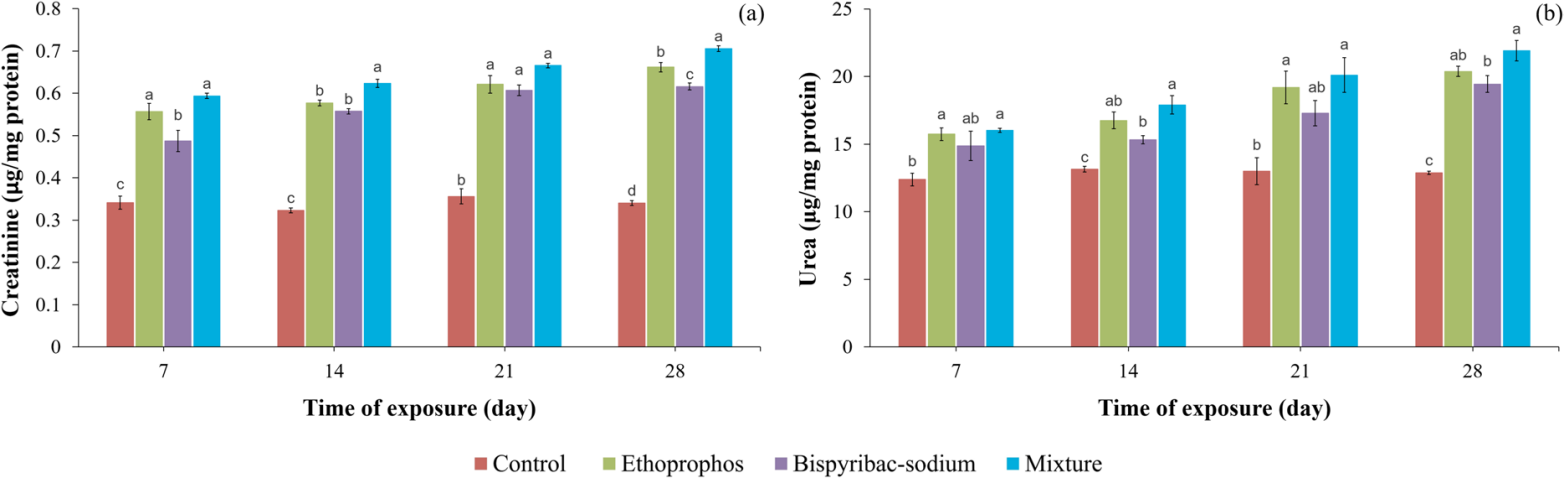
Level of creatinine (**a**) and urea (**b**) of Nile tilapia (*Oreochromis niloticus*) treated with ethoprophos, bispyribac-sodium, and their combination at different exposure times. Values are the mean ± SE, 10 animals (3 replicates per each) were used for each treatment. Superscript different letters (a, b, c) indicate statistically significant differences (*p* ≤ 0.05) between treatments at each exposure time

The tested compounds at all periods caused a marked elevation (*p* ≤ 0.05) in creatinine and urea levels (Fig. 3a and b). Fish poisoned for 28 days with ethoprophos, bispyribac-sodium, and their mixture exhibited increase in the creatinine levels by 1.95, 1.81, and 2.08 times, respectively, while in the level of urea counted by 20.38, 19.44, and 21.91 µg urea/mg protein, respectively, compared to the control value (12.89 µg urea/mg protein).

### Genotoxic marker

**Fig. 4:**
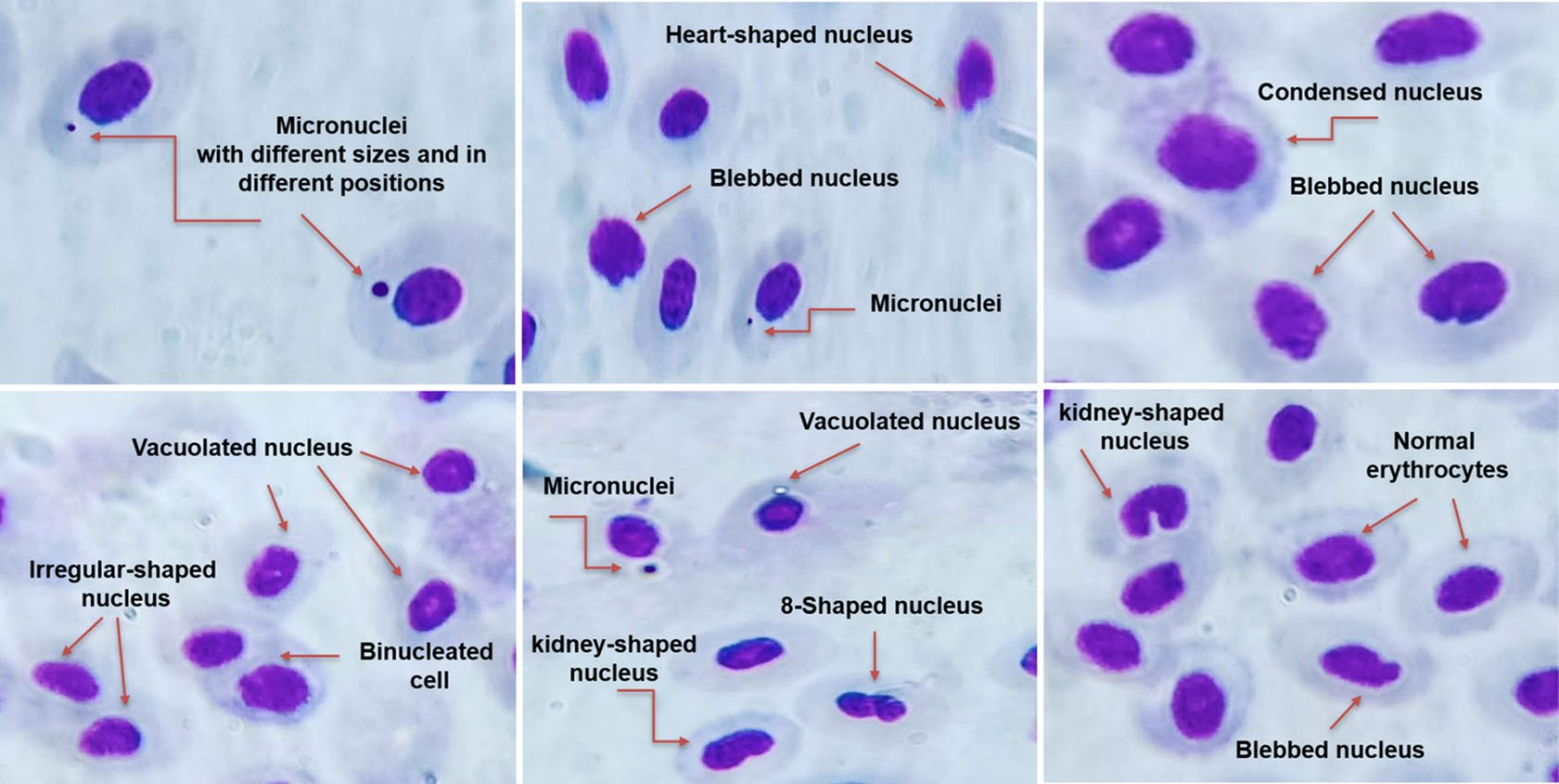
Erythrocytic nuclear abnormalities in *Oreochromis niloticus* after exposure to ethoprophos, bispyribac-sodium, and their combination for 4 weeks: micronuclei, vacuolated nucleus, irregular-shaped nucleus, binucleated cell, 8-shaped nuclei, kidney-shaped nucleus, heart-shaped nucleus, blebbed nucleus, and condensed nucleus

In this study, several erythrocyte nuclear abnormalities were apparent in fish blood smears, including micronuclei, binuclei, vacuolated nucleus, irregular-shaped nucleus, 8-shaped nucleus, kidney-shaped nucleus, heart-shaped nucleus, blebbed nucleus, and condensed nucleus (Fig. 4). The frequency of MN in exposed fish was significantly (*p* ≤ 0.05) increased by time and reached to maximum induction after 28 days of pesticide exposure by ratio 77.43, 39.34, and 82.04 of control value for ethoprophos, bispyribac-sodium, and their mixture, respectively (Table 1).

**Table 1:** Effect of sublethal exposure of ethoprophos, bispyribac-sodium and their combination on MN of Nile tilapia (*Oreochromis niloticus*) at different time intervals

Treatment	Frequency of MN (%)			
	Time of exposure (day)			
	7	14	21	28
Control	0.167 ± 0.09^d^	0.2 ± 0.15^d^	0.1 ± 0.10^c^	0.167 ± 0.12^d^
Ethoprophos	6.4 ± 0.21^b^	8.33 ± 0.19^b^	10.73 ± 0.33^a^	12.93 ± 0.20^b^
Bispyribac-sodium	3.5 ± 0.12^c^	4.77 ± 0.15^c^	5.47 ± 0.23^b^	6.57 ± 0.20^c^
Mixture	7.1 ± 0.06^a^	9.03 ± 0.03^a^	11.4 ± 0.21^a^	13.7 ± 0.23^a^

Values are the mean ± SE, 10 animals (3 replicates per each) were used for each treatment

The mean value in each column followed by the same letter is not significantly different (*p* ≤ 0.05)

### Interaction of pesticide mixture

Exposure to the mixture of pesticides showed both synergism and antagonism interactions (Table 2). Synergism and antagonism were considered when the observed effect of the mixture was higher or lower than the predicted effect, respectively. The negative effect of mixture exposure (observed) after 7, 14, 21, and 28 days on the activity of AChE and ALP was higher than predicted, giving synergy ratios equal to 0.97, 0.80, 0.63, and 0.65, and 0.61, 0.87, 0.93, and 0.90, respectively. Activity of AST and ALT was also more negatively affected by exposure to the mixture than expected after 7 days of exposure. In contrast, the response of other biomarkers changed to antagonism.

**Table 2:** The joint action of different markers measured in *Oreochromis niloticus* after exposure to sublethal concentration of ethoprophos/bispyribac-sodium mixture at different time intervals

Parameter	Days of treatment											
	7			14			21			28		
	Pred.	Obs.	Ratio	Pred.	Obs.	Ratio	Pred.	Obs.	Ratio	Pred.	Obs.	Ratio
AChE	0.38	0.39	0.97 (S)	0.24	0.30	0.80 (S)	0.17	0.27	0.63 (S)	0.11	0.17	0.65 (S)
ALP	0.70	1.15	0.61 (S)	1.04	1.20	0.87 (S)	1.39	1.49	0.93 (S)	1.99	2.20	0.90 (S)
AST	1.23	1.33	0.92 (S)	1.52	1.25	1.21 (A)	2.06	1.40	1.47 (A)	2.21	1.94	1.14 (A)
ALT	1.36	1.43	0.95 (S)	2.13	1.69	1.26 (A)	3.23	2.25	1.44 (A)	4.05	2.64	1.53 (A)
Urea	1.52	1.29	1.18 (A)	1.48	1.36	1.09 (A)	1.96	1.54	1.27 (A)	2.38	1.70	1.40 (A)
Creatinine	2.33	1.74	1.34 (A)	3.08	1.93	1.60 (A)	2.98	1.87	1.60 (A)	3.52	2.07	1.70 (A)
MN	806.4	42.6	18.93 (A)	993.1	45.2	21.97 (A)	5867.6	114	51.47 (A)	3057.4	82.2	37.20 (A)

Pred. means predicted effect and Obs. means observed effect. Ratio equivalent to predicted effect/observed effect

(S) means synergistic interaction and (A) means antagonism

## Discussion

There are a great number of substances in the environment that pose a threat to the quality of aquatic systems. One of the main sources of contamination by metals, sewage, pesticides, medications, and other toxins is anthropogenic activity. Therefore, it is necessary to evaluate a set of biomarkers at different levels of biological organization to indicate the health status of fish and water quality. Biochemical parameters are used as important biomarkers in toxicity research. Any deviation in biochemical parameters indicates some sort of disturbance in the animal’s homeostasis and could potentially deteriorate its health.

AChE, an important enzyme in the function of the nervous system, has been studied in many organisms. It involves nerve impulse transmission at synapses and breakdowns the neurotransmitter acetylcholine (Lionetto et al. 2013). The enzyme can be a target for several xenobiotics, and its inhibition by these substances leads to impaired nerve function. AChE is employed as a particular indicator to measure neurotoxic alterations (Mena et al. 2014;Topal et al. 2017). AChE activity is prevalent and frequently employed biomarker in the field of environmental toxicology, mostly with the aim of evaluating the impacts of contaminants at low concentrations (Paduraru et al. 2021).

Inhibition of AChE activity in the current study at sublethal concentrations of ethoprophos, bispyribac-sodium, and their combination demonstrates that the investigated compounds had a neurotoxic effect on the treated fish. The mechanisms of inhibition may involve the inability of AChE to hydrolyze acetylcholine. This can lead to the accumulation of ACh at synapses, which may affect the function of the nervous system, survival, growth, feeding, and reproductive behavior of fish subjected to various contaminants (Dutta and Arends 2003; Ibrahim et al. 2024), as well as disrupting energy metabolism and reducing overall stress tolerance (Bui-Nguyen et al. 2015). Several researchers have found decreased brain AChE activity in fish exposed to various pesticides, particularly organophosphates and carbamates (Lionetto et al. 2013; Kirici 2022), as well as many other classes of pesticides, such as herbicides (Gholami-Seyedkolaei et al. 2013). In the current study, the AChE inhibition patterns were more pronounced with ethoprophos than bispyribac-sodium, which may be due to the fact that ethoprophos is an alkyl phosphorodithioate compound that can directly inhibit AChE function via the phosphorylation of serine residue at the catalytic domain of the enzyme, while bispyribac-sodium does not have the specific chemical groups that contribute to AChE inhibition; the effect of bispyribac-sodium on AChE may be attributed to the indirect effect of benzoate present in its chemical structure, and the ethoprophos and bispyribac-sodium may cause synergistic effects on AChE inhibition. The inhibition of AChE in the brain of Nile tilapia exposed to a low concentration (96 μg/L) of ethoprophos was parallel to previously reported effects of ethoprophos on *Atractosteus tropicus* at 50 and 400 μg/L for 96 h **(**Torres et al. 2012**)** and on *Astyanax aeneus* at 0.25, 0.5, and 1 mg/L for 96 h (Mena et al. 2014) and at 0.014 mg/L for 48 h (Sandoval-Herrera et al. 2019).

Bispyribac-sodium has a different mode of action and is considered a potential acetolactate synthase inhibitor in plants. The detected AChE inhibition by the sublethal concentration (1.28 µg/L) of bispyribac-sodium in Nile tilapia brain indicates that bispyribac-sodium may have a non-specific neurotoxic effect. In addition, high oxidative stress caused by exposure to this herbicide may be responsible for decreased AChE activity. Few researchers have noted that bispyribac-sodium inhibited AChE of *Cyprinus carpio* fish subjected to commercial herbicide bispyribac-sodium (Nominee®, SC) after 72 days (Toni et al. 2010). Likewise, Lajmanovich et al. (2013) indicated that a commercial formulation bispyribac-sodium individually, and in mixture with commercial formulation glyphosate caused inhibition of AChE activity of *Rhinella arenarum* at 48 h and they found that the inhibition of AChE activity was higher in bispyribac-sodium exposed animals than in the mixture.

Considering the intensive use of these types of pesticides (organophosphates and herbicides) in Egyptian agriculture (Hathout et al. 2022), AChE appears as an ideal indicator to assess the impacts of agricultural pollution in the region. It would also be helpful to establish a link between pesticide usage, environmental residue detection, and harmful consequences. Moreover, the percent AChE inhibition values obtained in the treated fish with no mortality observed at the tested concentration, support further evidence of this biomarker as an early caution indicator.

The liver is essential for the metabolism and breakdown of contaminants like pesticides. Therefore, liver testing can be used to identify xenobiotic toxicity. The best sensitive and specific indicators of liver function are ALP, AST, and ALT, which are often utilized in the clinical diagnosis of hepatic functions and act as markers of liver health. The liver’s metabolism depends on the zinc-containing metalloenzyme ALP, which is an essential and vital enzyme that readily changes following pesticide poisoning. It is responsible for metabolism, detoxification and the manufacture of energy macromolecules necessary for a number of crucial processes (Ogueji et al. 2020). In present study, the elevation of hepatic ALP activity in *O. niloticus* may be liable for the adverse effects of the assessed pesticides on liver cells, which may lead to liver injury (Temiz and Kargın 2024). According to the findings of Ragab et al. (2024), a significant increase in serum ALP activity of *Clarias gariepinus* treated with the sublethal concentrations of bispyribac-sodium (0.11 and 0.37 mg/L) for 14 and 28 days was reported. Similar findings of substantial increase in ALP activity were reported by several authors in *O. niloticus* exposed to various pesticides (Fathy et al. 2019; de Bem Matos et al. 2022; Kanu et al. 2023).

Transaminases are found in two isozymes: AST, which is primarily a mitochondrial enzyme, and ALT, which is a crucial cytoplasmic enzyme. They help convert proteins into energy for liver cells and play an important role in the synthesis and deamination of amino acids under stressful conditions to meet the body’s high energy needs (Hassanein 2004). In the current study, the elevated ALT and AST activities in Nile tilapia exposed to all substances may be attributed to the changes in protein and carbohydrate metabolism under stress conditions. The increased activity of transaminases can be considered as a measure of the compensatory mechanism of metabolic disorders, meaning that a rise in ALT and AST activities suggests that aspartate and alanine are being mobilized for gluconeogenesis to produce glucose in order to withstand stress (Kumar et al. 2014). In addition, the elevated AST and ALT activities, which are a consequence of oxidative stress induced by the studied pesticides **(**Toni et al. 2010; Li et al. 2021**)**, indicate an enhance in protein catabolism and liver cell damage or liver cirrhosis (Ogueji et al. 2020). Our observations are in harmony with the data published by Ragab et al. (2024) who found induction in the activity of AST and ALT in *Clarias gariepinus* exposed to 0.11 and 0.37 mg/L of bispyribac-sodium for 14 and 28 days. Similar increases in AST and ALT activities were observed in *O. niloticus* when exposed to bispyribac-sodium (Fathy et al. 2019), chlorpyrifos (Majumder and Kaviraj 2019; Kanu et al. 2023**)** and glyphosate **(**Abdelmagid et al. 2023).

AST and ALT are regarded as pertinent stress indicators and are frequently utilized in the diagnosis of fish illnesses and the detection of tissue damage produced by environmental contamination (Kumar et al. 2014; Banaee et al. 2024). Fish liver is mainly rich in transaminases since it is the main site of food interconversion and is considered an indicator of water pollution (Gül et al. 2004). Liver is the place of biotransformation by which a poisonous substance has been changed to a less dangerous form to decrease toxicity. However, this will harm the tissue membrane and cells of liver and cause hepatotoxicity.

In the current investigation, ethoprophos, bispyribac-sodium, or their combination is converted into their metabolites after entering the biological system. Both the chemical and its metabolites have the potential to attach to cellular macromolecules and interact with free amino groups of proteins, causing the macromolecules to lose their physiological activities or inducing the production of additional hazardous metabolites by hepatocytes. These substances cause hepatotoxicity and stimulate the production of liver enzymes. Additionally, they could harm cells by covalent attachment to enzymes, nucleic acids and proteins. Damage of cellular components may play a significant role in mortality of liver cells. Since ALP and transaminases are involved in the processes of metabolism, detoxification, and biosynthesis of energy macromolecules for various critical functions, the deviation of these enzymes from normal values indicates biochemical disorders, tissue damage, increased permeability of cell membranes, and cellular functions (Ambali et al. 2007; Banaee et al. 2023; Temiz and Kargın 2024).

The kidney is an excretory organ that removes metabolic waste and performs an osmoregulatory function in fish. The kidney is the critical target organ for toxic substances that cause a range of renal toxic effects affecting tubular cells and glomerulus (Eissa and Zidan 2010; Gowda et al. 2010). Creatinine and urea are metabolic parameters that can be used as biomarkers to assess physiological renal function. Creatinine is a non-protein nitrogenous waste product resulting from the metabolism of creatine in skeletal muscle. It diffuses freely through body fluids, is filtered by the kidneys, and is subsequently excreted in the urine. Urea is generated in the liver by deamination of amino acids, and then it is transported by blood to the kidneys where it is eliminated with urine.

According to our data, increased level of creatinine and urea in Nile tilapia exposed to sublethal concentration of tested toxicants is often indicative of a reduction in the kidney’s capacity to filter waste materials and eliminate them in the urine. Kidney failure may be due to the accumulation of parent substances and/or their metabolites in the kidney, which may impair the reabsorption capacity of the proximal tubules (Li et al. 2015) and decrease glomerular filtration rate (Wang et al. 2013). In addition, impairment of the renal secretory function due to the decrease in renal blood flow has been associated with higher urea concentrations. As well as, enhanced protein catabolism and/or the transformation of ammonia to urea due to more production of the enzyme arginase, that is involved in urea production. Our results are supported by the work of Fathy et al. (2019), who observed an increase in the level of creatinine and urea in Nile tilapia exposed to bispyribac-sodium (1/100 field rate concentration) for 96 h. Other studies also showed the same results in fish: *Cyprinus carpio* after exposure to profenofos and imidacloprid (Abdel Rahman et al. 2020; Banaee et al. 2024), *O. niloticus* after subject to glyphosate (Abdelmagid et al. 2023) and *Tor putitora* after exposure to chlorpyrifos and dichlorvos alone and mixed (Kunwar et al. 2022).

The coupling of chromosomal damage with biochemical markers can provide a complete indication of the influence of chemical contaminants on Nile tilapia. Fish genotoxicity biomarkers are valuable endpoints for environmental risk assessment. The MN test as an indicator of accumulated genetic injury during the life of the cells is one of the most suitable methods to identify integrated response to the complex mixture of pollutants. It is also one of the most commonly utilized methods for determining structural and numerical chromosomal changes in different systems in vitro and in vivo. It is created from chromosome fragments or entire chromosomes that lag during cell division as a result of centromere injury, lack of a centromere or a defect in cytokinesis. The current results showed that the tested compounds increased the frequency of erythrocyte nuclear abnormalities, including MN, in fish blood in a time-dependent manner. Blood is a reflection of pathophysiology, and different red blood cell abnormalities are considered as leading biomarkers for assessing pollution-related adverse effects and demonstrate the link between chronic health effects and mutagenicity (Bolognesi and Hayashi 2011). The increased frequency of MN suggests that genotoxic and mutagenic effects are present in this experiment, which may be due to increased production of caspase-activated DNase, leading to cleavage of nuclear and cytoskeletal proteins and aneuploidy (Hussain et al. 2014).

As this is the first study to investigate the genotoxic potential of ethoprophos on Nile tilapia, hence it is not possible to compare our findings with similar studies. Whereas, little information is available on the genotoxic effects of bispyribac-sodium in fish. Pradhan et al. (2020) observed a rise in the incidence of micronuclei in peripheral erythrocytes of *Clarias batrachus* with increasing concentrations of bispyribac-sodium (0.027, 0.036, and 0.054 mg/L) after 20, 25, and 30 days of exposure. An increased frequency of MN was also found in specimens of *O. niloticus* collected from three different sections in Burullus Lake, which is one of the major disposal areas for agricultural drainage water in Egypt (El Nahas et al. 2017). There are some articles including MN induction in different species of fish exposed to various pesticides (Hong et al. 2018; Amaeze et al. 2020; Ayanda et al. 2021). The results of this study highlight the importance of the MN assay as an early biological marker of fish exposure to different pollutants in the aquatic environment. However, the MN test can vary depending on pollution type, fish species, age, sex, cell life cycle, diet, and weathering conditions (Bolognesi and Hayashi 2011).

In natural conditions, compounds coexist with other chemicals. This fact may lead to interactions between them, and the complication of the interactions is influenced by variances in chemical characteristics, mechanisms of action and the existence of detoxifying enzymes in the body (Bhagat et al. 2021). Moreover, the combination may affect multiple systems simultaneously, thereby altering the predicted toxicity. It is unclear how ethoprophos and bispyribac-sodium interact in Nile tilapia and what the potential joint effects might be. These pollutants may affect the markers studied depending on the duration of exposure. To the best of our knowledge, the present study provides the first experimental data on the biological effects of ethoprophos and bispyribac-sodium mixtures in Nile tilapia. The mixture of the two tested pesticides at sublethal concentrations resulted in more antagonistic than synergistic effects. Joint toxicity was characterized as synergism in AChE and ALP at all time of exposure and in AST and ALT at 7 days of exposure, while other biomarkers response changed to antagonism. It is likely that the joint effects of the two tested pesticides were due to interplay between their respective modes of action and their effects on physiological processes. Additional investigation is needed to know the actual cause of the reported response. Previous studies have shown that bispyribac-sodium individually, and in mixture with glyphosate caused inhibition of AChE activity of *Rhinella arenarum*, this inhibition was higher in bispyribac-sodium exposed animals than in the mixture (Lajmanovich et al. 2013). Likewise, zebrafish, *Danio rerio*, treated with Roundup-chlorpyrifos mixtures suffered more DNA damage than controls, but less than fish treated with chlorpyrifos alone (Falfushynska et al. 2022). Additionally, Bacchetta et al. (2014) studied the effects of sublethal concentrations of endosulfan, lambda-cyhalothrin, and their combination for 96 h on a panel of biomarkers including hematological parameters, transaminases and alkaline phosphatase activities and oxidative damage biomarkers in *Piaractus mesopotamicus*. They found that the mixture produced the most significant effects. Furthermore, Banaee et al. (2024) found that exposure of *Cyprinus carpio* to the combination of imidacloprid and chlorpyrifos had synergistic effects on some oxidative and biochemical biomarkers of fish than the single compound. Moreover, combined exposure to mancozeb and metalaxyl showed synergistic effects in the transcription of genes that involved in detoxification and biochemical indicators in zebrafish (Banaee et al. 2023).

Compared with chemical monitoring, biological effects-based pollutant assessment is more advantageous in terms of cost, sensitivity, and predictive capacity. Biomarkers can demonstrate the effects of pollutants on the environment and can be used to monitor fish health and as early warning indicators of environmental risks. In a physiologically healthy animal, AChE activity, markers of liver and kidney function, and genotoxic parameter are in a normal steady state. Many environmental pollutants, including pesticides, can produce ROS in the biological system. Excess ROS can cause DNA damage, lipid peroxidation and protein oxidation in tissues, leading to neurotoxicity, hepatotoxicity, nephrotoxicity, and genotoxicity of organism.

The use of a variety of sensitive and simple assays as endpoints provides a complete picture and better understanding of pollutant exposure. Overall, the results of this work indicate the effectiveness of the measured parameters and their application in identifying and assessing the effects of low concentrations of tested chemicals in the aquatic environment. Thanks to biomarkers that can identify many problems in both medicine and the environment. This approach offers a number of specific benefits, including reduced scope and cost of research, early detection of hazards to vulnerable populations, and the ability to quickly preventative and/or corrective actions in damaged ecosystems.

## Conclusion

The in vivo effect of the sublethal concentration of ethoprophos and bispyribac-sodium applied individually or together for different time intervals (7, 14, 21, and 28 days) changed several parameters in Nile tilapia. All three treatments were found to inhibit brain AChE activity, increase the activity of liver function enzymes (ALP, AST, and ALT), kidney function parameters (creatinine and urea), and the frequency of MN. The results show that the mixture of the two tested pesticides has more antagonistic than synergistic effects. It is also important to highlight the need for in-depth studies related to pesticide mixtures, as they are widely used in agriculture. These compounds should be tested in non-target organisms such as fish using sensitive methods to determine their sublethal effects that may lead to irreversible changes causing permanent damage to fish populations. Due to the lack of fish studies assessing effects following exposure to ethoprophos and bispyribac-sodium mixtures and given the realistic exposure situation in agricultural waters, these results provide important information for future studies.

## Data Availability

All data analyzed during this study are included in this article. The raw data that support the findings of this study are available on request.
